# The alterations of airway and intestine microbiota in asthma: a systematic review and meta-analysis

**DOI:** 10.3389/fimmu.2025.1675124

**Published:** 2025-09-29

**Authors:** Wenpei Li, Kezhen Lu, Jisen Tang, Yanyu Chen, Yifan Lu, Xiaoyan Hu, Haixing Zhu, Yun Feng

**Affiliations:** ^1^ Department of Respiratory and Critical Care Medicine, Ruijin Hospital Affiliated Shanghai Jiao Tong University School of Medicine, Shanghai, China; ^2^ Shanghai Jiao Tong University School of Medicine, Shanghai, China

**Keywords:** human airway microbiome, human intestine microbiome, alpha diversity, beta diversity, relative abundance, asthma, meta-analysis

## Abstract

**Background:**

Emerging evidence highlights notable differences in microbial ecology between individuals with asthma and healthy controls (HC). This meta-analysis aims to compile data on microbial diversity indices in the airway and intestinal microbiota of both groups for comparative analysis.

**Methods:**

We conducted a thorough systematic search of literature in PubMed, Embase, the Web of Science, and the Cochrane Library to find English-language studies focused on airway and intestinal microbiota in asthma, published from May 16, 2020 to May 16, 2025. We extracted data regarding microbial diversity indices to facilitate comparisons between the asthma group and HC.

**Results:**

26 studies were included in this systematic review. Our analysis revealed no significant differences in alpha diversity between the two participant groups; however, beta diversity exhibited significant differences in 9 of the studies reviewed.

**Conclusion:**

Our meta-analysis did not confirm the hypothesis that asthma shows lower alpha diversity than HC. To enhance understanding and inform future diagnostic and therapeutic approaches, further studies should be conducted with larger sample sizes and more robust methodologies.

**Systematic review registration:**

https://www.crd.york.ac.uk/prospero/, identifier CRD420251113790.

## 1 Introduction

Asthma is a chronic airway inflammatory disease with underlying immunological disorders. It affects over 300 million people worldwide, and its pathogenesis involves complicated interplays among immune and microbial factors (1). Recently, with the swift development of microbiomics technology, the roles of the respiratory and intestinal microbiota in the occurrence and development of asthma have attracted increasing attention. Studies have shown that changes in the composition and function of the microbiota may be involved in the pathogenesis of asthma by regulating host immune responses, maintaining mucosal barrier integrity, and influencing metabolic pathways (2). For example, specific probiotic interventions may palliate asthma symptoms (3). Early antibiotic use and dietary changes may affect immune development through the intestinal microbiota and increase the risk of asthma (4). Microbes can also interact with the host immune system through structural ligands (such as lipopolysaccharides) and metabolic products (such as short-chain fatty acids (SCFAs)), influencing the development and progression of the disease (5). In recent years, many *in vitro* and animal studies have revealed the functional role of the airway and intestine microbiota in asthma. Alterations in the respiratory microbiota, such as the enrichment of *Hemophilus* and *Moraxella*, which can activate neutrophil and eosinophil inflammation through Toll-like receptor (TLR) signaling, have been demonstrated to promote the asthma phenotype in mice models (6). It is worth noting that there is a two-way communication system between the intestine and the lungs, namely the “gut-lung axis”. Animal studies have shown that the composition of the gut microbiota and its metabolites, such as SCFAs, can migrate to the airway through the blood circulation and regulate local immune homeostasis (5). In turn, respiratory inhaled microbial exposure may also affect the composition and function of intestinal microbiota and further regulate systemic immune response (7). For instance, evidence suggests that exposure to bacterial pathogens colonizing the respiratory tract (e.g., *Streptococcus pneumoniae*) can translocate to the gut via swallowing or immune cell-mediated transport, thereby altering the gut microbiota structure and influencing distal mucosal immunity (8).

Although a great number of studies have explored the association between the microbiota and asthma, the results of different studies are inconsistent. This may be due to differences in sample sources (such as the respiratory tract and the intestine), sequencing methods, population characteristics (such as age and geographical region), and asthma phenotypes. Therefore, systematically integrating existing evidence to clarify the specific patterns of microbiota changes in asthma and their underlying mechanisms is of scientific and clinical significance.

This meta-analysis aims to comprehensively assess differences in respiratory and intestinal microbiota composition and diversity between asthma patients and HC in an attempt to discover possible diagnostic and therapeutic roles of microbiota in asthma management. Through a systematic review of existing studies, we hope to establish a theoretical foundation for microbiota intervention strategies in asthma and propose future research directions.

## 2 Materials and methods

The searches were carried out following the revised 2020 guidelines of the Preferred Reporting Items for Systematic Reviews and Meta-Analyses (PRISMA) statement and checklist (9).

### 2.1 Data sources and search strategy

We conducted a systematic review utilizing four databases: PubMed, Web of Science, Embase, and The Cochrane Library. This investigation concentrated solely on original research articles involving human subjects, published in English between May 16, 2020 and May 16, 2025. The focus was on literature related to the bacterial microbiota in asthma, specifically within two anatomical regions: the airway and the intestine. Our search strategy employed medical subject headings (MeSH) alongside relevant free-text terms to ensure comprehensive coverage of pertinent studies. The MeSH terms utilized were ‘Microbiota’ [Mesh] and ‘Asthma’[Mesh]. The free-text terms related to’Microbiota’ [Mesh] are ‘Microbiotas’, ‘Microbial Community’, ‘Community, Microbial’, ‘Microbial Communities’, ‘Microbial Community Composition’, ‘Community Composition, Microbial’, ‘Composition, Microbial Community’, ‘Microbial Community Compositions’, ‘Microbiome’, ‘Microbiomes’, ‘Human Microbiome’, ‘Human Microbiomes’, ‘Microbiome, Human’, ‘Microbial Community Structure’, ‘Community Structure, Microbial’ and ‘Microbial Community Structures’. The free-text terms related to’Asthma’[Mesh] are ‘Asthmas’, ‘Asthma, Bronchial’ and ‘Bronchial Asthma’. Detailed search strings can be found in **Supplementary Table 1**.

### 2.2 Eligibility criteria

The titles and abstracts were examined independently by two researchers. Any differences in their assessments were settled by reaching a consensus with a third researcher. To maintain uniformity in screening criteria, all three investigators participated in standardized training before initiating the formal literature review. The criteria for including studies are as follows (1): Participants must be adults over 18 years old diagnosed with asthma and healthy controls (HC) (2); The study design should be observational, categorizing as either case-control or cross-sectional, and published in English (3); The article must discuss microbial characteristics found in either the airways or the intestines of individuals with asthma (4); It should involve either metagenomic sequencing or 16S rRNA sequencing analysis (5); The study must provide reports on microbial diversity indices, either within the main article or in its **Supplementary Materials**, with specific numerical values available for extraction from relevant figures or tables; and (6) There should be at least one control group comprising healthy individuals or patients with stable disease, as well as a minimum of one case group made up of patients with stable disease or those experiencing exacerbations.

Studies will be excluded based on the following criteria (1): Research involving non-human subjects or individuals younger than 18 (2); Studies that do not clearly present microbial diversity indices or lack extractable data from microbial diversity charts (3); Research without a comparative control group; and (4) Article formats including abstracts, case reports, expert opinions, reviews, letters, or editorials.

All selected studies adhered to the stated eligibility criteria, with a main emphasis on evaluating bacterial alpha diversity in various samples from both asthma patients and healthy controls (HC). This assessment included metrics such as the Chao1 index, Shannon index, and Simpson index. Initially, articles were reviewed through their titles and abstracts, followed by a comprehensive evaluation of full texts to identify those that ultimately fulfilled the inclusion criteria. The final selection was achieved through consensus among all the authors.

### 2.3 Data extraction and synthesis

The required data from the included studies were gathered by two researchers and documented in a custom-designed Excel spreadsheet. The extracted information included the following variables (1): Study details (title, name of the first author, publication year, journal, study design type, and sample type) (2); Population characteristics (sample size, various disease states of asthma, and age) (3); Community-level assessments of microbial composition in different bodily sites (airway and intestine), with a primary focus on alpha diversity followed by beta diversity. Taxonomic findings at both the phylum and genus levels, including bacterial relative abundance, were also noted (4); Sequencing details (NGS sequencing method and the specific amplification region of 16S rRNA). Quantitative measures for alpha diversity were derived using Get Data Graph Digitizer software when it was necessary to extract specific data from graphical representations.

### 2.4 Quality assessment and risk of bias

Study quality was evaluated with the Newcastle-Ottawa Scale (NOS), a widely accepted instrument for gauging the risk of bias in observational research. The scale allocates points across three domains: participant selection (up to 4 points), between-group comparability (up to 2 points), and ascertainment of outcomes (up to 3 points), allowing a maximum total of 9 points. Research earning 7–9 points was judged to be of high methodological rigor, scores of 4–6 signified moderate quality, and totals below 4 indicated low quality. Detailed ratings for each study are displayed in **Supplementary Figure 1** and **Supplementary Table 2**.

### 2.5 Statistical analysis

Between-group differences in microbial diversity were quantified as standardized mean differences (SMDs) and 95% confidence intervals (CIs) using RevMan 5.4. When continuous outcomes were reported as means with standard deviations (SD), these values were used directly; when medians and inter-quartile ranges were supplied instead, standard formulas were applied to approximate the corresponding mean and SD before pooling the data (10, 11). For categorical outcomes—such as the relative abundance of individual taxa—data from all eligible studies were synthesized. Fixed-effect and random-effect meta-analyses were run in parallel, with forest plots produced to illustrate between-group differences in community composition. Robustness of the pooled estimates was explored with leave-one-out sensitivity checks, and small-study effects were screened using Egger’s regression (Stata 16.0).

## 3 Results

### 3.1 Literature search and study characteristics

**Figure 1:**
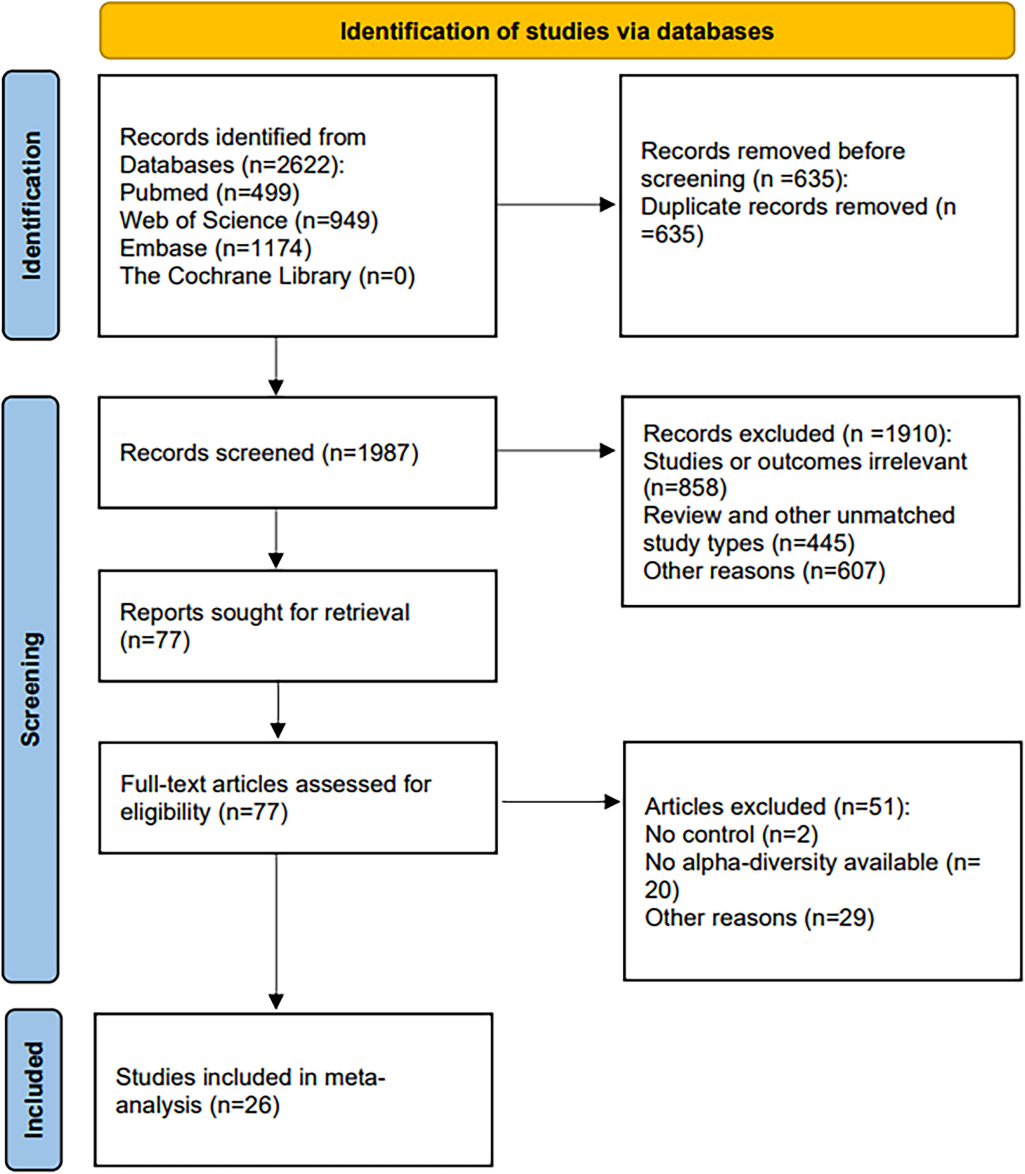
PRISMA flow diagram of selected studies for inclusion.

Our initial search across four databases—PubMed (499), Web of Science (949), Embase (1,174), and the Cochrane Library (0)—yielded 2,622 records. After de-duplication with EndNote and supplementary hand-checking, 1,987 unique citations remained. Title-and-abstract screening then removed 1,910 records: 858 were off-topic, 445 used an ineligible design, and 607 were excluded for other reasons. Full texts of the remaining 77 manuscripts were evaluated, leading to the inclusion of 26 studies in the systematic review; the PRISMA flow diagram in **Figure 1** details every step.

**Table 1** summarizes the key attributes of the 26 studies retained for the meta-analysis.

**Table 1:** Characteristics of each study included in the meta-analysis.

Study references	Site	Design	Type of sample	NGS sequencing	Healthy	Asthma
Rana et al.	Nasal cavity (airway)	Case-control	Nasopharyngeal swab	Illumina, 16S rRNA sequencing	5	10
	Intestine	Case-control	Fecal		5	15
Gu et al.	Intestine	Case-control	Fecal	16S rRNA sequencing	18	28
Zou et al.	Intestine	Cross-sectional	Fecal	16S rRNA sequencing	20	47
Kullberg et al.	Intestine	Case-control	Fecal	16S rRNA sequencing	1460	172
Tjitske S. R. van Engelen et al.	Intestine	Cross-sectional	Fecal	16S rRNA sequencing	–	20
	Airway	Cross-sectional	BALF		–	20
Ham et al.	Airway	Case-control	Sputum	16S rRNA sequencing	23	74
	Intestine	Case-control	Fecal		23	74
Turek et al.	Oropharynx (airway)	Cross-sectional	Posterior oropharyngeal swabs	16S rRNA sequencing	529	529
Ahmad R. Alsayed et al.	Airway	NR	Sputum	Illumina	–	8
Al Bataineh et al.	Airway	Case-control	Sputum	16S rRNA sequencing	10	10
Yang et al.	Airway	Case-control	Sputum	16S rRNA sequencing	–	45
Versi et al.	Airway	Case-control	Sputum	Illumina, Whole genome sequencing	24	23
Wang et al.	Airway	Case-control	Sputum	16S rRNA sequencing	21	63
Tanabe et al.	Airway	Cross-sectional	Sputum	16S rRNA sequencing	–	10
Chen et al.	Nasal cavity (airway)	Case-control	Nasal rinse	16S rRNA sequencing	20	30
Zhang et al.	Airway	Case-control	NP and IS	16S rRNA sequencing	16	23
Diver et al.	Airway	Cohort	Sputum	Illumina	–	140
Pinto et al.	Oropharynx (airway)	Cross-sectional	Subgingival biofilm	16S rRNA sequencing	10	20
Rick et al.	Airway	Case-control	Sputum	ITS2 sequencing	14	83
Wang et al.	Airway	Cross-sectional	Sputum	16S rRNA sequencing	–	80
Marcos Pérez-Losada et al.	Nasal cavity (airway)	Cross-sectional	Nasopharyngeal swab	Illumina, ITS1 and ITS2 sequencing	125	12
Perez-Garcia et al.	Airway	Case-control	Saliva samples, nasal and pharyngeal swabs	16S rRNA sequencing	–	250
Al-Ramahi et al	Airway	Case-control	Sputum	Illumina, 16S rRNA sequencing	27	27
Xu et al.	Oropharynx (airway)	Cross-sectional	Oropharyngeal swab	Illumina, ITS sequencing	10	8
Marcos Pérez-Losada et al.	Nasal cavity (airway)	Cross-sectional	Nasopharyngeal swab	16S rRNA sequencing	99	12

NR, not reported; “-”, none; BALF, Bronchoalveolar lavage fluid; NP, nasopharyngeal; IS, induced sputum.

To describe microbial communities within the airway and intestine, investigators most often reported alpha diversity metrics. When these indices were pooled across studies, marked heterogeneity emerged; variability appeared driven by differences in cohort size, specimen source, and sampling protocols (**Supplementary Tables 3**–**5**).

### 3.2 Quality assessment

All manuscripts were graded with the Newcastle-Ottawa Scale. Following review, nine were rated high-quality, fifteen moderate-quality, and two low-quality; the precise NOS ratings for each report are mapped in **Supplementary Figure 1**.

### 3.3 Comparisons of alpha diversity between HC and asthma

**Figure 2:**
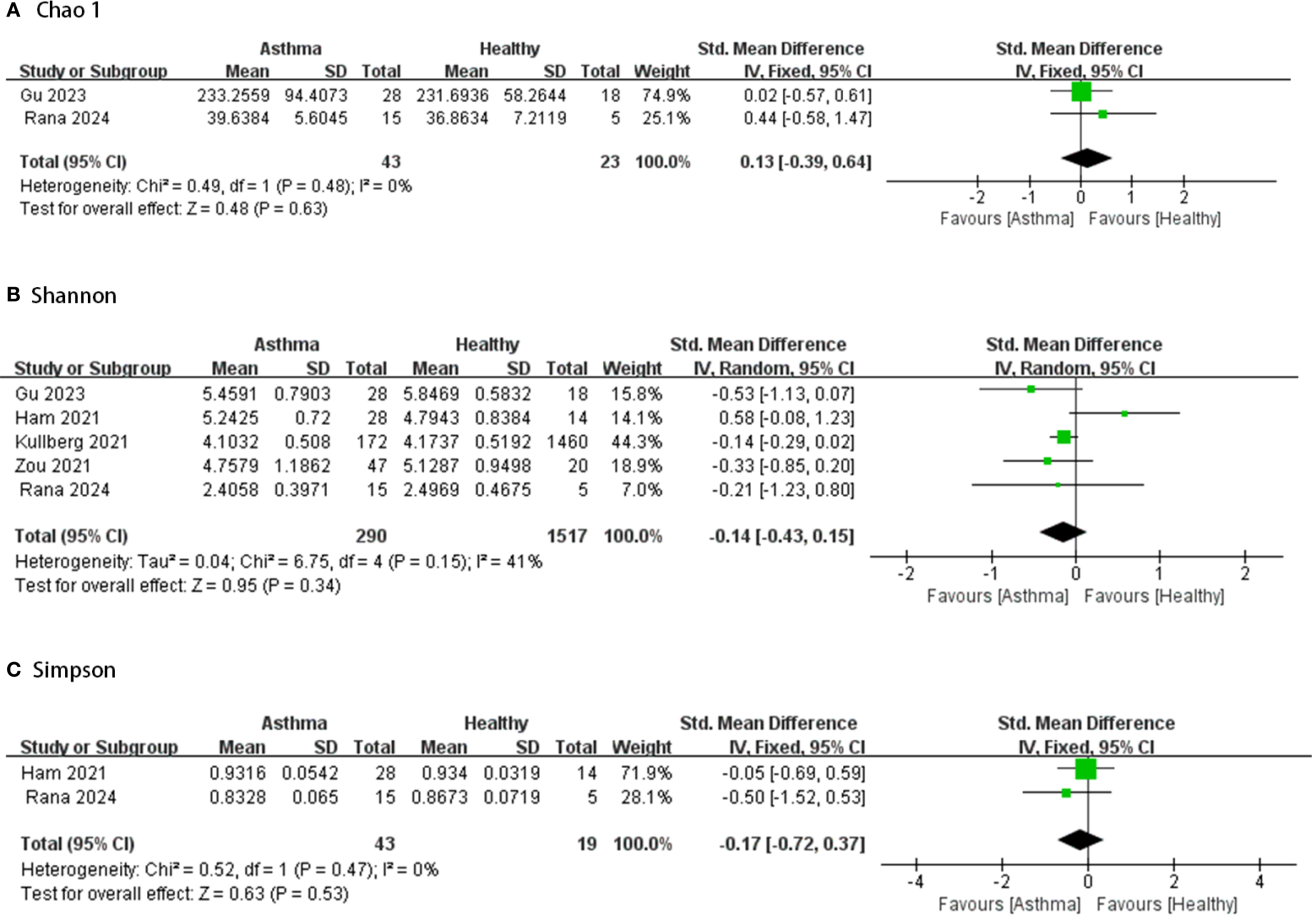
Forest plot of randomized controlled trials comparing the intestinal microbial alpha-diversity between HC and asthma. **(A)** Chao 1; **(B)** Shannon; **(C)** Simpson.

**Figure 3:**
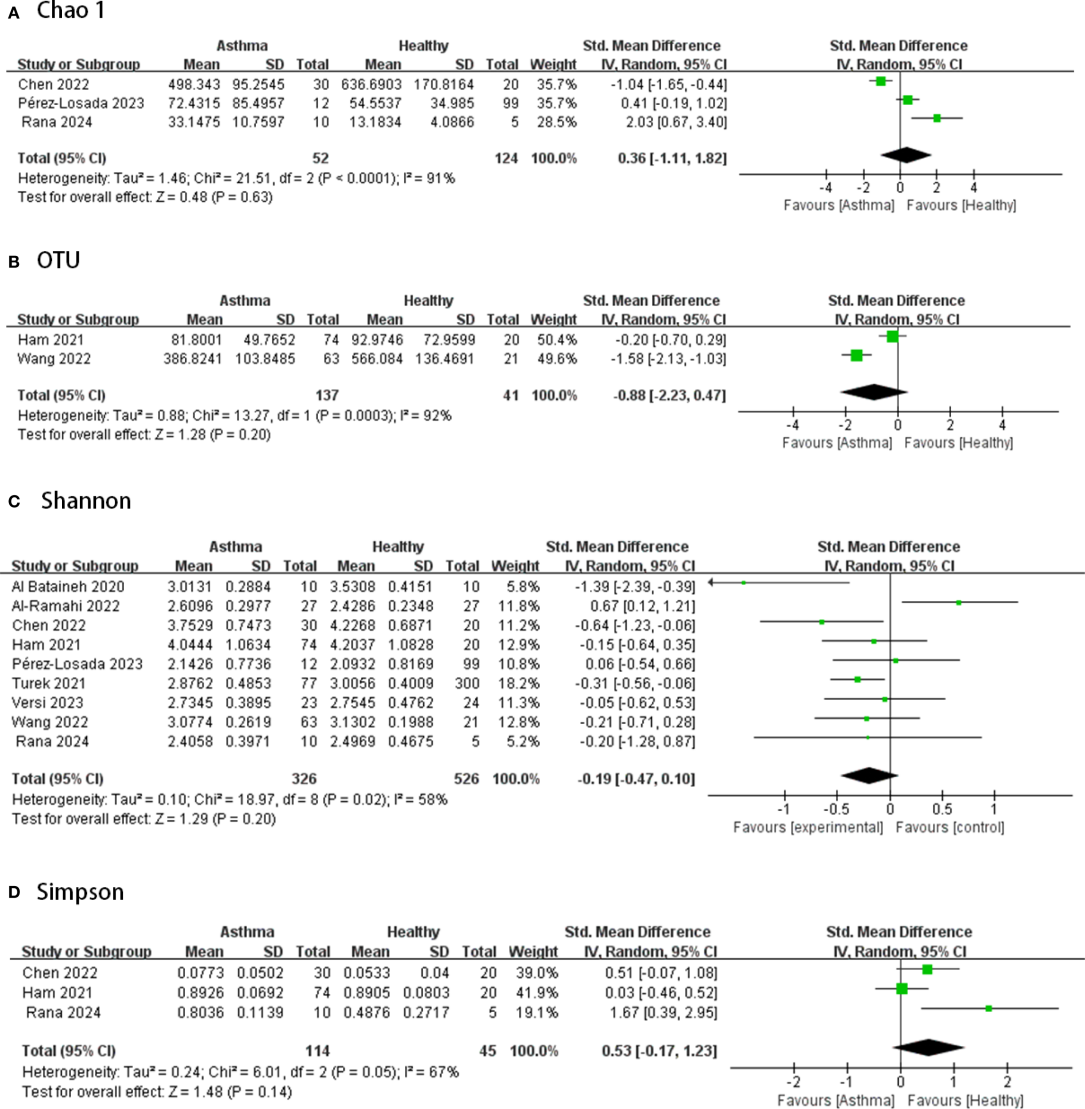
Forest plot of randomized controlled trials comparing the airway microbial alpha-diversity between HC and asthma. **(A)** Chao 1; **(B)** OTU; **(C)** Shannon; **(D)** Simpson.

**Figure 4:**
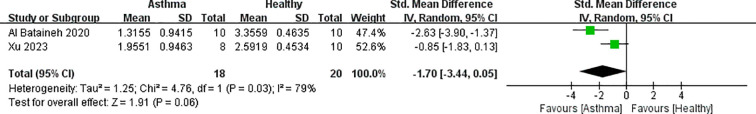
Forest plot of randomized controlled trials comparing the airway fungal alpha-diversity (Shannon) between HC and asthma.

**Figures 2**–**4** display the pooled standardized mean differences and 95% confidence intervals contrasting case and control groups. Among the five intestine-microbiota publications, two quantified diversities with the Chao1 index, five used the Shannon, and two employed Simpson. Within the nine respiratory-microbiota studies, three reported Chao1, three reported observed OTUs, all nine supplied Shannon indices, and two also included Simpson. Two additional studies, identified during screening, compared airway fungal alpha diversity (Shannon) between healthy and asthmatic participants (12, 13).

#### 3.3.1 Intestinal microbiota

Comparing the intestinal microbial alpha-diversity between HC and asthma, based on **Figure 2**, it is clear that most studies showed lower alpha diversity index in the asthma group compared to the healthy group. Only a few studies reported higher alpha diversity index in the intervention group (14–16). However, in most of the comparisons, the intergroup alpha diversity differences between healthy and asthmatic intestinal microbes were not significant. Five studies provided Chao1, Shannon and/or Simpson indices of intestinal microbiota in samples from 290 asthmatic and 1,517 healthy cases. None showed great heterogeneity (I^2^<50%) and there were no significant differences between groups (95% CIs all spanning zero).

#### 3.3.2 Airway bacterial microbiota

According to **Figure 3**, in terms of airway microbiota diversity, the nine studies included 326 asthmatic and 526 healthy samples, for which the data from all studies of the airway microbiota demonstrated a high degree of heterogeneity (I^2^ > 50%, p ≤0.05), and there were no significant differences in alpha diversity between the groups (p>0.05, all spanning zero).

#### 3.3.3 Airway fungal microbiota

According to **Figure 4**, two studies provided airway fungal flora data for 18 asthmatic and 20 healthy samples. The forest plot revealed a high degree of heterogeneity (I^2^ = 79%, p = 0.03) and no significant difference in alpha diversity between groups (p = 0.06, 95% CI spanning zero).

### 3.4 Comparisons of beta diversity between HC and asthma

Of all the included articles, 18 studies reported beta diversity (**Supplementary Table 6**). For intestinal microbiota, several studies did not observe significant differences in beta diversity between asthma and healthy groups (14, 17). Another study found a significant difference in the overall microbial composition between asthma and HC (p = 0.004) (18).

With regard to airway microbes, several studies have found no significant differences in beta diversity between disease phenotypes (19, 20); others have found significant differences in beta diversity between asthma and health (12, 21–24). Still another study has shown that significant differences in beta diversity exist between eosinophilic and non-eosinophilic inflammatory phenotypes (25). However, another study reached the opposite conclusion: microbial composition was not associated with inflammatory subsets based on sputum or blood eosinophils (26). This may be due to the difficulty of ensuring airway microbial samples are not contaminated during extraction.

### 3.5 Differences in microbial taxa abundance

In the intestinal tracts, most included studies claimed that gut microbial compositions were significantly different between asthmatic and HC. *Firmicutes* (41%) showed higher relative abundance in non-asthmatic subjects (23). One study found that *Akkermansia muciniphila* was significantly reduced in asthmatics (15). In symptomatic eosinophilic asthma, the relative abundances of *Lachnospiraceae* and *Oscillospiraceae* significantly decreased and *Bacteroidetes* increased in the gut microbiota. In this case, *Lachnospiraceae* was negatively correlated with indicators of type 2 inflammation and lung function decline, while *Enterobacteriaceae* and *Prevotella* was positively associated with type 2 inflammation and lung function decline, respectively (14). However, in an urban, large-sized and ethnically diverse cohort, one study reported no prominent differences in fecal microbiota composition were discovered in adult asthmatics when compared to non-asthmatics (17).

In terms of the airway microbiota, the majority of studies agree that there is an increase in *Bacteroidetes* in the asthma group compared to the control group (14, 15, 21). It has also been reported that the relative abundance of *Proteobacteria* is higher in asthmatics than in non-asthmatics (30% and 17% respectively; p = 0.044). The pathogen *Hemophilus influenzae* is also found to be in higher abundance in asthmatics. Another study found that only two taxa (*Neisseria* and *Roseburia*’s OTUs) showed an increased abundance in asthmatic airways, with *Neisseria*’s OTUs enriched (accounting for 4% of the population readings) and showed a 2-fold increase, which is consistent with the increase in *Proteobacteria* observed consistently through comparisons of asthmatic and normal airways. In asthmatic subjects, 84 OTUs were relatively low in abundance, including *Leptotrichia*, *Selenomonas*, *Megasphaera*, and *Capnocytophaga*. Some of the more common genera, such as *Actinobacteria*, *Prevotella* and *Veillonella*, were also less abundantly represented in asthmatics (19). Findings are summarized in **Supplementary Table 7**.

It has been suggested that differences in the lung phenotypes of airway microbiota are associated with and may influence asthma, particularly the inflammatory phenotypes (21). It has been proposed that alterations in the respiratory microbiota are not merely epiphenomena but actively participate in driving or modulating inflammatory processes, thereby influencing asthma severity, clinical stability, and treatment responsiveness. For instance, Taylor et al. demonstrated that a microbiota dominated by *Hemophilus* or *Moraxella* is closely associated with allergic sensitization, elevated biomarkers of Th2-mediated inflammation (such as FeNO and blood eosinophil counts), and increased exacerbation risk (27). Similarly, Durack et al. revealed that during asthma exacerbations, the diversity of the airway microbiota sharply declines, accompanied by an expansion of pro-inflammatory bacterial taxa. Conversely, microbial diversity partially recovers as clinical stability is restored. These dynamic shifts in the microbiota composition directly reflect disease activity, suggesting an active role in asthma pathogenesis (28).

### 3.6 Risk of bias and sensitivity analysis

Forest plots comparing asthma and healthy-control groups revealed marked heterogeneity across most pooled outcomes; the only exceptions were the Chao1 and Simpson indices derived from intestinal samples, which exhibited no discernible heterogeneity.

Across the 9 airway-microbiota studies depicted in the forest plots, Shannon’s index was the most commonly applied alpha diversity metric. To probe the heterogeneity observed in these nine datasets, we performed a leave-one-out sensitivity analysis, iteratively excluding individual studies to assess the robustness and precision of the pooled estimate (**Supplementary Figure 2**). After removing each study in turn, the effect sizes of two studies changed significantly. However, the shifts in effect sizes were in different directions, and most changes in effect sizes were within reasonable limits. Overall, the results remained largely stable.

The reliability of the pooled Shannon-index findings for the airway microbiota in asthma was further scrutinized with a funnel plot and Egger’s regression. The results showed that the Egger’s test correlation p-value for the asthma studies exceeded 0.05 (p = 0.947) and the regression line intercept was close to zero (**Supplementary Figure 3**), suggesting a lack of evidence supporting publication bias. Considering that only nine studies were used as the sample for the test, its negative result does not completely rule out publication bias. Further observation of the funnel plot (**Supplementary Figure 4**), which has good symmetry, reinforcing confidence that the pooled estimate is unlikely to be distorted by selective reporting.

## 4 Discussion

While previous reviews have described changes in the airway or intestinal microbiota in isolation, we report the first meta-analysis to compare airway and intestine microbial diversity between individuals with asthma and healthy controls (HC) with an integrative approach, capturing the latest evidence with improved sequencing technologies and analytical methods, which has not been comprehensively done in previous reviews on this topic. Based on existing literature, we hypothesized that asthmatic patients exhibit reduced alpha diversity in airway and intestine microbiota compared to HC. Contrary to our initial hypothesis and findings from many existing studies, we found no significant overall difference in microbial alpha diversity between the two groups. Although most individual studies pointed toward diminished alpha diversity in asthma, pooled estimates did not confirm a statistically significant reduction, most likely because of the small overall sample. Moreover, 13 additional investigations on airway or intestine microbiota met our inclusion criteria but lacked the necessary groupings or data for forest-plot synthesis. Given the restricted evidence base—especially the paucity of studies on the intestinal microbiota—findings derived from the meta-analytic comparisons should be interpreted cautiously.

Alpha diversity is often considered critical for microbial communities, but it is oversimplistic to categorize high-diversity communities as intrinsically “superior” or somehow more valuable than low-diversity communities (29). Although respiratory-microbiota studies displayed pronounced heterogeneity, leave-one-out testing indicated that the pooled Shannon index remained stable across the nine included datasets. To further elucidate the sources of heterogeneity across the included airway-microbiota studies, several factors were identified, including sampling sites, sequencing techniques, data processing pipelines, and patient characteristics. For example, regarding airway samples, sputum was the most commonly used specimen type; however, exceptions were observed. Bronchoalveolar lavage fluid (BALF) was employed in the study by Tjitske S. R. van Engelen et al., while nasal or oral swabs were utilized in several other investigations. Additionally, differences in sequencing methodologies contributed substantially to the observed heterogeneity. Whereas the majority of studies relied on 16S rRNA sequencing, whole genome sequencing was implemented in the research conducted by Versi et al. Given that these factors may affect the comparability of diversity indices across studies, the results of the leave-one-out analysis enhance confidence in the comparative conclusions.

Taken together with the other forest-plot comparisons, these findings neither confirm nor refute a difference in overall microbial composition between asthma and HC, implying that no single, clear biomarker reliably distinguishes the two groups at present. Beta diversity serves as an indicator assessing similarities in microbial community composition among different sample groups by focusing on variations in microbial community structure across samples (30). Principal-coordinate (PCoA) and principal-component (PCA) ordinations were extracted from 18 eligible studies to evaluate beta diversity contrasts. Half of these reports revealed discernible separation in community structure between asthma and healthy-control specimens, whereas the remainder detected no such divergence (**Supplementary Table 6**). The inconsistent results in beta diversity comparisons underscore the complexity of microbial community structural changes in asthma. Given the use of divergent statistical methods for assessing beta diversity and the presence of inconsistent findings across the included studies, it is not feasible to establish consistent patterns or identify taxa that are universally associated.

In addition, data from included studies were also used to compare taxon-level abundance across populations, and consistent alterations in the relative abundance of several bacterial taxa were observed. Notably, most of the studies agree that asthmatics contain potential pathogens in their bodies, suggesting that their lungs have a unique microbial composition. In respiratory diseases, infections mainly lead to acute exacerbations. Most studies showed an increase in the relative abundance of *Bacteroidetes*, *Proteobacteria* and *Hemophilus influenzae*, as well as a decrease in *Firmicutes*, in asthma patients compared to controls (14, 15, 21). *Hemophilus* is present in the nasopharynx of humans. Asthma-related airway remodeling and hyperresponsiveness alter local structure and immunity, fostering niches where *Hemophilus* can persist. In the gut, *Bacteroidetes* and *Firmicutes* dominate dietary fiber fermentation, generating short-chain fatty acids (SCFAs) that modulate host responses linked to allergic inflammation through epigenetic mechanisms (31). They reduce eosinophil functions, including adhesion, migration, and survival, accompanied by an elevation in global H3 acetylation (32). A key benefit of epigenetic regulation of SCFAs is the enhancement of regulatory T cell (Treg) differentiation and function. Specifically, the SCFA butyrate acts as a histone deacetylase (HDAC) inhibitor, leading to increased histone acetylation critical for Treg differentiation and function, and enhancing Treg suppressive capacity, which is crucial for maintaining immune tolerance and dampening Th2-driven inflammatory responses characteristic of asthma. These beneficial effects of SCFAs on the immune system was shown first in mice and then in humans (33, 34). Moreover, SCFA-mediated reduction of murine mast cell activity was also attributed to their roles on histone modifications (35). The interplay between nutrition, SCFA and epigenetic modification offers a compelling mechanistic framework to explain how those compositional changes might contribute to disease pathogenesis (36)*. Proteobacteria* contains a wide range of potential pathogens, and these bacteria produce endotoxins (lipopolysaccharide, LPS), proteases, and toxins that directly irritate and damage the airway epithelium. Abnormally elevated abundance of *Proteobacteria* (especially in the intestine) is often seen as a marker of overall dysbiosis. The role of *Firmicutes* in asthma is bi-directional - encompassing both protective effects and pro-inflammatory potential. Among them, some are commensal flora producing SCFAs, whereas *streptococci* colonize the human nasopharynx and produce a variety of virulence factors, including polysaccharide capsule, which contributes to the development of respiratory diseases such as pneumonia (37).

In addition, a reduced abundance of representative species of *Actinobacteria*, *Prevotella*, and *Veillonella* has been observed in asthma (19). *Veillonella* is a gram-negative anaerobic bacterium that belongs to the normal oral commensal flora; however, its exact role remains elusive (38). It has been noted that during asthma, the abundance of *Prevotella* decreases while pathogenic bacteria proliferate; however, the potential homeostatic function of *Prevotella* in healthy lungs remains largely unknown (39). The aggregated evidence points toward *Actinobacteria*, *Prevotella*, and *Veillonella* as taxa that may exert protective effects in either healthy or diseased states.

Our meta-analysis compared microbial diversity between different disease states of asthma and healthy HC. After screening 26 publications (13 of which, totaling 2,677 participants, entered forest-plot analyses), our study suggests that asthma is not universally characterized by a decrease in microbial alpha diversity but is associated with distinct compositional alterations in both the airway and intestine microbiota. Interpretation is, however, tempered by four limitations: (i) the intestinal microbiota literature remains sparse, limiting statistical power; (ii) most datasets derived from 16S rRNA sequencing, precluding species-level resolution and offering limited raw data for re-analysis; (iii) pooled airway-microbiota analyses displayed substantial heterogeneity, although sensitivity analysis confirmed the stability of the main findings; (iv) restricting the review to last five years omits earlier research on airway and intestine microbiota in asthma, creating a risk of selection bias; (v) the limited number of studies utilizing each sample type precluded stratification of results by specific sampling sites; (vi) potential confounding variables, including antibiotic usage, smoking status, corticosteroid treatment, and sequencing platform, may introduce bias and affect the interpretation of outcomes; and (vii) approximating means and standard deviations from reported medians and inter-quartile ranges may have introduced minor inaccuracies in diversity estimates.

## 5 Conclusion

In conclusion, our review does not support the widely held expectation that asthma is accompanied by a universal reduction in alpha diversity; but it reveals clear disparities in beta diversity between asthmatic and healthy individuals across both airway and intestinal niches. By integrating these body sites with quantitative meta-analytic synthesis of diversity indices for the first time, we highlight the complexity of microbial perturbations in asthma and underscore the need for larger, multi-center studies employing standardized protocols in sampling, sequencing, and analysis. Future longitudinal research is also essential to capture temporal dynamics of microbial communities before robust biomarker profiles can be established for guiding precision therapies.

## Data Availability

The datasets presented in this study can be found in online repositories. The names of the repository/repositories and accession number(s) can be found in the article/**Supplementary Material**.
