# Supplementary material for: The alterations of airway and intestine microbiota in asthma: a systematic review and meta-analysis
Source: Front Immunol. 2025 Sep 29;16:1675124. doi: 10.3389/fimmu.2025.1675124 (PMC12515863; doi:10.3389/fimmu.2025.1675124)
Supplement: Supplementary file 1 [file Table1.docx]

Supplementary Material

**Supplementary Table 1.** Systematic search detail.

**Supplementary Figure 1.** Quality score of included articles calculated using the NOS.

**Supplementary Table 2.** Quality assessment of the included studies using the Newcastle-Ottawa Scale.

**Supplementary Table 3.** Alpha-diversity index of intestinal microbiota in HC and asthma.

**Supplementary Table 4.** Alpha-diversity index of airway microbiota among HC and asthma.

**Supplementary Table 5.** Fungal alpha-diversity index of airway microbiota among HC and asthma.

**Supplementary Table 6.** Summary of beta diversity assessments in the included studies.

**Supplementary Table 7.** Summary of differences in microbial taxa abundance in the included studies.

**Supplementary Figure 2.** Sensitivity analysis for the Shannon index results for the asthma airway microbiomes of included studies.

**Supplementary Figure 3.** Egger's test for the Shannon index results for the asthma airway microbiomes of included studies.

**Supplementary Figure 4.** Funnel plot for the Shannon index results for the asthma airway microbiomes of included studies.

**Supplementary Table 1. Systematic search detail.**

- 1. **Pubmed search stratergy for 499**

| **Search** | **Hits** |
| --- | --- |
| 1#  (((((((((((((((((((((("Microbiota"[Mesh]) OR (Microbiota[Title/Abstract])) OR (Microbiotas[Title/Abstract])) OR (Microbial Community[Title/Abstract])) OR (Community, Microbial[Title/Abstract])) OR (Microbial Communities[Title/Abstract])) OR (Microbial Community Composition[Title/Abstract])) OR (Community Composition, Microbial[Title/Abstract])) OR (Composition, Microbial Community[Title/Abstract])) OR (Microbial Community Compositions[Title/Abstract])) OR (Microbial Community Structure[Title/Abstract])) OR (Community Structure, Microbial[Title/Abstract])) OR (Microbial Community Structures[Title/Abstract])) OR (Microbiome[Title/Abstract])) OR (Microbiomes[Title/Abstract])) OR (Human Microbiome[Title/Abstract])) OR (Human Microbiomes[Title/Abstract])) OR (Microbiome, Human[Title/Abstract])) OR (Flora[Title/Abstract])) OR (Microflora[Title/Abstract])) OR (Dysbiosis[Title/Abstract])) OR (Ecosystem[Title/Abstract])) OR (Bacteria[Title/Abstract]) | 797823 |
| 2#  **(((Asthma[Mesh]) OR (Asthmas[Title/Abstract])) OR (Asthma, Bronchial[Title/Abstract])) OR (Bronchial Asthma[Title/Abstract])** | 153273 |
| **3#：1# AND 2#**  ((((((((((((((((((((((("Microbiota"[Mesh]) OR (Microbiota[Title/Abstract])) OR (Microbiotas[Title/Abstract])) OR (Microbial Community[Title/Abstract])) OR (Community, Microbial[Title/Abstract])) OR (Microbial Communities[Title/Abstract])) OR (Microbial Community Composition[Title/Abstract])) OR (Community Composition, Microbial[Title/Abstract])) OR (Composition, Microbial Community[Title/Abstract])) OR (Microbial Community Compositions[Title/Abstract])) OR (Microbial Community Structure[Title/Abstract])) OR (Community Structure, Microbial[Title/Abstract])) OR (Microbial Community Structures[Title/Abstract])) OR (Microbiome[Title/Abstract])) OR (Microbiomes[Title/Abstract])) OR (Human Microbiome[Title/Abstract])) OR (Human Microbiomes[Title/Abstract])) OR (Microbiome, Human[Title/Abstract])) OR (Flora[Title/Abstract])) OR (Microflora[Title/Abstract])) OR (Dysbiosis[Title/Abstract])) OR (Ecosystem[Title/Abstract])) OR (Bacteria[Title/Abstract])) AND ((((Asthma[Mesh]) OR (Asthmas[Title/Abstract])) OR (Asthma, Bronchial[Title/Abstract])) OR (Bronchial Asthma[Title/Abstract])) | 1705 |
| Limits：English | 1536 |
| Limits：Humans | 1332 |
| Limits：in the last 5 years | **499** |

- 1. **Web of Science search stratergy for 949**

| **Search** | **Hits** |
| --- | --- |
| (TS=(Microbiota) OR AB=(Microbiota OR Microbiotas OR Microbial Community OR Community, Microbial OR Microbial Communities OR Microbial Community Composition OR Community Composition, Microbial OR Composition, Microbial Community OR Microbial Community Compositions OR Microbial Community Structure OR Community Structure, Microbial OR Microbial Community Structures OR Microbiome OR Microbiomes OR Human Microbiome OR Human Microbiomes OR Microbiome, Human OR Flora OR Microflora OR Dysbiosis OR Ecosystem OR Bacteria)) and Preprint Citation Index (Exclude – Database) | 2509793 |
| TS=(Asthma) OR AB=(Asthmas OR Asthma, Bronchial OR Bronchial Asthma) and Preprint Citation Index (Exclude – Database) | 466368 |
| #1 AND #2 and Preprint Citation Index (Exclude – Database) | 9647 |
| Limits：English (Languages) | 9105 |
| Limits：2025 or 2024 or 2023 or 2022 or 2021 (Publication Years) | 3064 |
| Limits：Article (Document Types) | 1958 |
| Limits：Respiratory System (Research Areas) | **949** |

- 1. **Embase search stratergy for 1174**

| **Search** | **Hits** |
| --- | --- |
| 1#  microbiota:ab,ti OR microbiotas:ab,ti OR 'microbial community':ab,ti OR 'community, microbial':ab,ti OR 'microbial communities':ab,ti OR 'microbial community composition':ab,ti OR 'community composition, microbial':ab,ti OR 'composition, microbial community':ab,ti OR 'microbial community compositions':ab,ti OR 'microbial community structure':ab,ti OR 'community structure, microbial':ab,ti OR 'microbial community structures':ab,ti OR microbiome:ab,ti OR microbiomes:ab,ti OR 'human microbiome':ab,ti OR 'human microbiomes':ab,ti OR 'microbiome, human':ab,ti OR flora:ab,ti OR microflora:ab,ti OR dysbiosis:ab,ti OR ecosystem:ab,ti OR bacteria:ab,ti | 877947 |
| 2#  'asthma':ab,ti OR 'asthmas':ab,ti OR 'asthma,bronchial':ab,ti OR 'bronchial asthma':ab,ti | 276492 |
| 3#  #1 AND #2 | 4661 |
| 4#  #3 AND [english]/lim AND [humans]/lim | 3530 |
| 5#  #4 AND (2021:py OR 2022:py OR 2023:py OR 2024:py OR 2025:py) | **1174** |

- 1. **Cochrane search stratergy for 0**

| **Search** | **Hits** |
| --- | --- |
| #1 MeSH descriptor: [Microbiota] explode all trees | 2482 |
| #2 (Microbiota or Microbiotas or Microbial Community or Community, Microbial or Microbial Communities or Microbial Community Composition or Community Composition, Microbial or Composition, Microbial Community or Microbial Community Compositions or Microbial Community Structure or Community Structure, Microbial or Microbial Community Structures or Microbiome or Microbiomes or Human Microbiome or Human Microbiomes or Microbiome, Human or Flora or Microflora or Dysbiosis or Ecosystem or Bacteria):ti,ab,kw (Word variations have been searched) | 31318 |
| #3 #1 or #2 | 31321 |
| #4 MeSH descriptor: [Asthma] explode all trees | 14654 |
| #5 (Asthma or Asthmas or Asthma, Bronchial or Bronchial Asthma):ti,ab,kw (Word variations have been searched) | 36385 |
| #6 #4 or #5 | 36394 |
| #7 #3 and #6 with Cochrane Library publication date Between May 2021 and May 2025 | **0** |

**
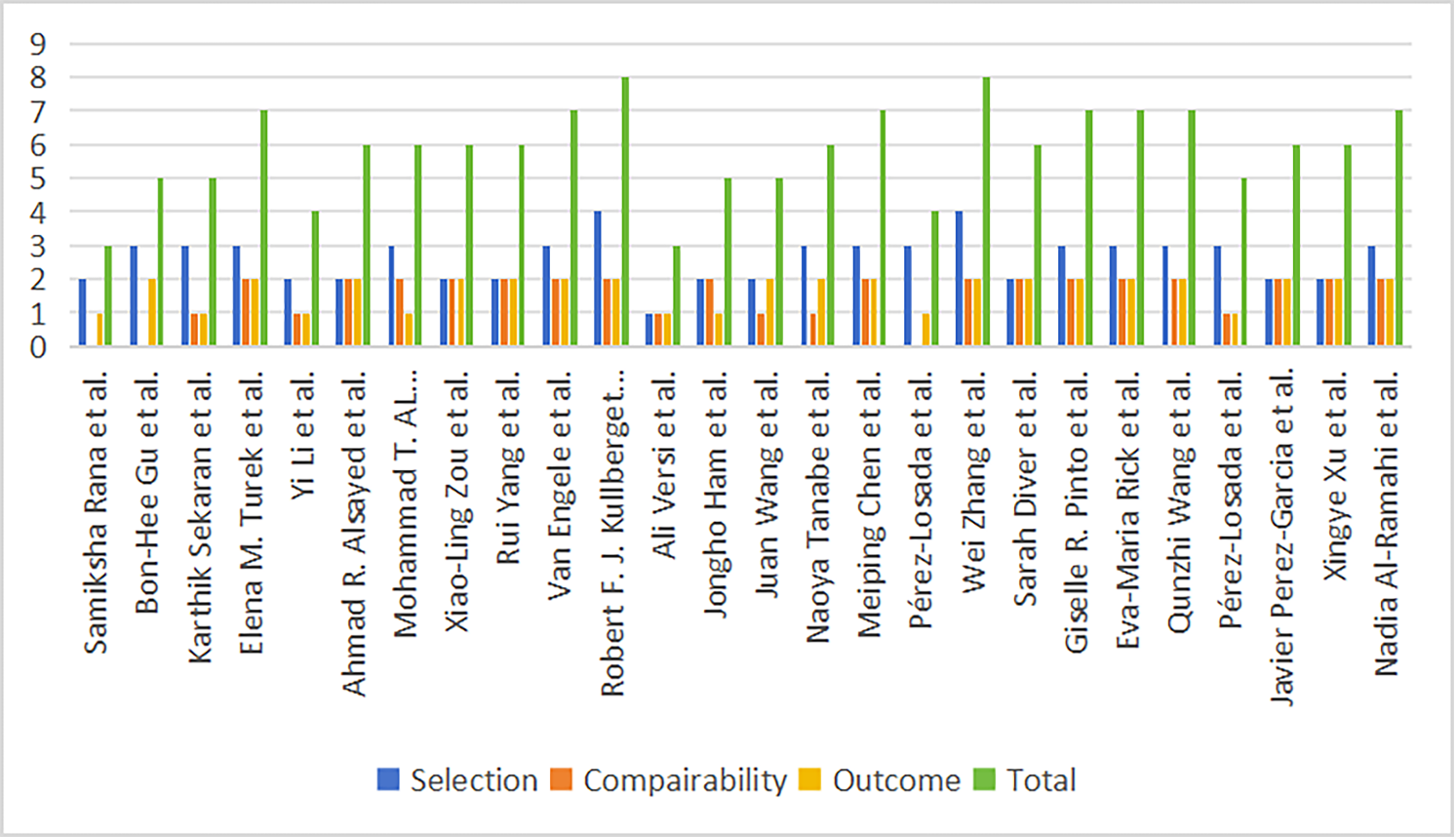
**

**Supplementary Figure 1. Quality score of included articles calculated using the NOS.**

**Supplementary Table 2.** **Quality assessment of the included studies using the Newcastle-Ottawa Scale.**

| **Study** | **Selection** | **Comparability** | **Outcome** | **Total** |
| --- | --- | --- | --- | --- |
| Samiksha Rana et al. | 2 | 0 | 1 | 3 |
| Bon-Hee Gu et al. | 3 | 0 | 2 | 5 |
| Karthik Sekaran et al. | 3 | 1 | 1 | 5 |
| Elena M. Turek et al. | 3 | 2 | 2 | 7 |
| Yi Li et al. | 2 | 1 | 1 | 4 |
| Ahmad R. Alsayed et al. | 2 | 2 | 2 | 6 |
| Mohammad T. AL Bataineh et al. | 3 | 2 | 1 | 6 |
| Xiao-Ling Zou et al. | 2 | 2 | 2 | 6 |
| Rui Yang et al. | 2 | 2 | 2 | 6 |
| Van Engele et al. | 3 | 2 | 2 | 7 |
| Robert F. J. Kullberg et al. | 4 | 2 | 2 | 8 |
| Ali Versi et al. | 1 | 1 | 1 | 3 |
| Jongho Ham et al. | 2 | 2 | 1 | 5 |
| Juan Wang et al. | 2 | 1 | 2 | 5 |
| Naoya Tanabe et al. | 3 | 1 | 2 | 6 |
| Meiping Chen et al. | 3 | 2 | 2 | 7 |
| Pérez-Losada et al. | 3 | 0 | 1 | 4 |
| Wei Zhang et al. | 4 | 2 | 2 | 8 |
| Sarah Diver et al. | 2 | 2 | 2 | 6 |
| Giselle R. Pinto et al. | 3 | 2 | 2 | 7 |
| Eva-Maria Rick et al. | 3 | 2 | 2 | 7 |
| Qunzhi Wang et al. | 3 | 2 | 2 | 7 |
| Pérez-Losada et al. | 3 | 1 | 1 | 5 |
| Javier Perez-Garcia et al. | 2 | 2 | 2 | 6 |
| Xingye Xu et al. | 2 | 2 | 2 | 6 |
| Nadia Al-Ramahi et al. | 3 | 2 | 2 | 7 |

**Supplementary Table 3.** Alpha-diversity index of intestinal microbiota in HC and asthma.

**Supplementary Table 4.** Alpha-diversity index of airway microbiota among HC and asthma.

**Supplementary Table 5.** Fungal alpha-diversity index of airway microbiota among HC and asthma.

The aforementioned three tables can be located in the supplementary Excel spreadsheet.

| Supplementary Table 6. Summary of beta diversity assessments in the included studies. | | | |
| --- | --- | --- | --- |
| **Study** | **β-diversity** | **Findings** | **Statistic value** |
| Gu et al. | Bray-Curtis distance | No significant differences in alpha and beta diversity were observed between the EA and control groups. | NR |
| Turek | Weighted and unweighted UniFrac distances | No significant differences in β-diversity were observed between disease phenotypes through PERMANOVA. | NR |
| AL Bataineh et al. | PCoA based on Bray-Curtis dissimilarities | Determined significant differences among different groups. | p < 0.0001 |
| Zou et al. | PLS-DA, PERMANOVA | The overall microbial composition was significantly different between asthmatic and healthy groups. | p = 0.004 |
| Ham et al. | PCoA based on weighted normalized UniFrac distance | The two groups did not differ in terms of the relative abundance of microbiome genera or species in the sputum. | NR |
| Kullberg et al. | unweighted and weighted Unifrac distances | No distinct clusters were observed when visualizing beta-diversity by principal coordinate analysis. | p = 0.97 |
| Versi et al. | Bray-Curtis dissimilarities | There was no shift in β-diversity of airway microbiome in severe asthma cases analyzed at the species or genus level. | NR |
| Wang et al. | ANOSIM based on Bray–Curtis and Jaccard distances | The asthma samples were significantly separated from healthy control based on community membership. | p < 0.001 |
| Chen et al. | PCoA and PLS-DA based on Quantitative Insights into Microbial Ecology (QIME, v1.80) | Evaluation of the general landscape of the nasal microbiome revealed similar bacterial communities at phylum and genus levels. | p < 0.05 |
| (continued) | | | |
| **Supplementary Table 6. (continued)** | | | |
| **Study** | **β-diversity** | **Findings** | **Statistic value** |
| Al-Ramahi et al. | One-way ANCOVA | Samples from the asthmatic patients have greater bacterial diversity than the samples from non-asthmatic subjects. | p < 0.05 |
| Xu et al. | PCoA, PERMANOVA | In the comparison of each pair of disease group and the HC group, LC group showed significant difference from the HC group. | p < 0.05 |
| Pérez-Losada et al. | PCoA based on Unifrac (unweighted and weighted), Bray–Curtis and Jaccard distances | Indices of microbial structure differed significantly between each of the respiratory disease groups and controls. | p < 0.011 |
| Li et al. | PERMANOVA with Bray-Curtis distance | A minimal difference of microbial communities in nasopharyngeal and salivary microbiome. | p = 0.85，  p = 0.4 |
| Yang et al. | NMDS plots based on Bray-Curtis distance, PERMANOVA | Within asthma and COPD, respectively, the fungal microbiota was dissimilar in subjects with different inflammatory phenotypes. | p = 0.001 |
| Zhang et al. | PERMANOVA based on Weighted-Unifrac distance | In induced sputum (IS) group, microbiota differed significantly between those with asthma and the HC group. | p < 0.001 |
| Diver et al. | PCoA based on Unifrac distance | No significant differences in different groups. | NR |
| Pinto et al. | PCoA based on Unifrac and Bray-Curtis distances | No significant differences in different groups. | NR |
| Rick et al. | Bray-Curtis dissimilarities | No significant differences between HC and asthmatic groups. | NR |

**Supplementary Table 7.** **Summary of differences in microbial taxa abundance in the included studies.**

| **Study** | **Site** | **Findings** |
| --- | --- | --- |
| Al-Ramahi et al. | Intestine | *Firmicutes* (41%) showed higher relative abundance in non-asthmatic subjects. |
| Rana et al. | Intestine | *Akkermansia muciniphila* was significantly reduced in asthmatics. |
| Gu et al. | Intestine | In symptomatic eosinophilic asthma, the relative abundances of *Lachnospiraceae* and *Oscillospiraceae* significantly decreased and *Bacteroidetes* increased in the gut microbiota. |
| Kullberg et al. | Intestine | In an urban, large-sized and ethnically diverse cohort, no prominent differences in fecal microbiota composition were discovered in adult asthmatics when compared to non-asthmatics. |
| Gu et al. | Airway | There is an increase in *Bacteroidetes* in the asthma group compared to the control group. The relative abundance of *Proteobacteria* is higher in asthmatics than in non-asthmatics (30% and 17% respectively; p = 0.044). The pathogen *Haemophilus influenzae* is also found to be in higher abundance in asthmatics. Additionally, two taxa (*Neisseria* and *Roseburia*) showed an increased abundance in asthmatic airways. |
| Rana et al. |  |  |
| Want et al. |  |  |
| Turek et al. | Airway | 84 OTUs were relatively low in abundance, including *Leptotrichia, Selenomonas, Megasphaera*, and *Capnocytophaga*. Some of the more common genera, such as *Actinobacteria, Prevotella* and *Veillonella*, were also less abundantly represented in asthmatics |

**
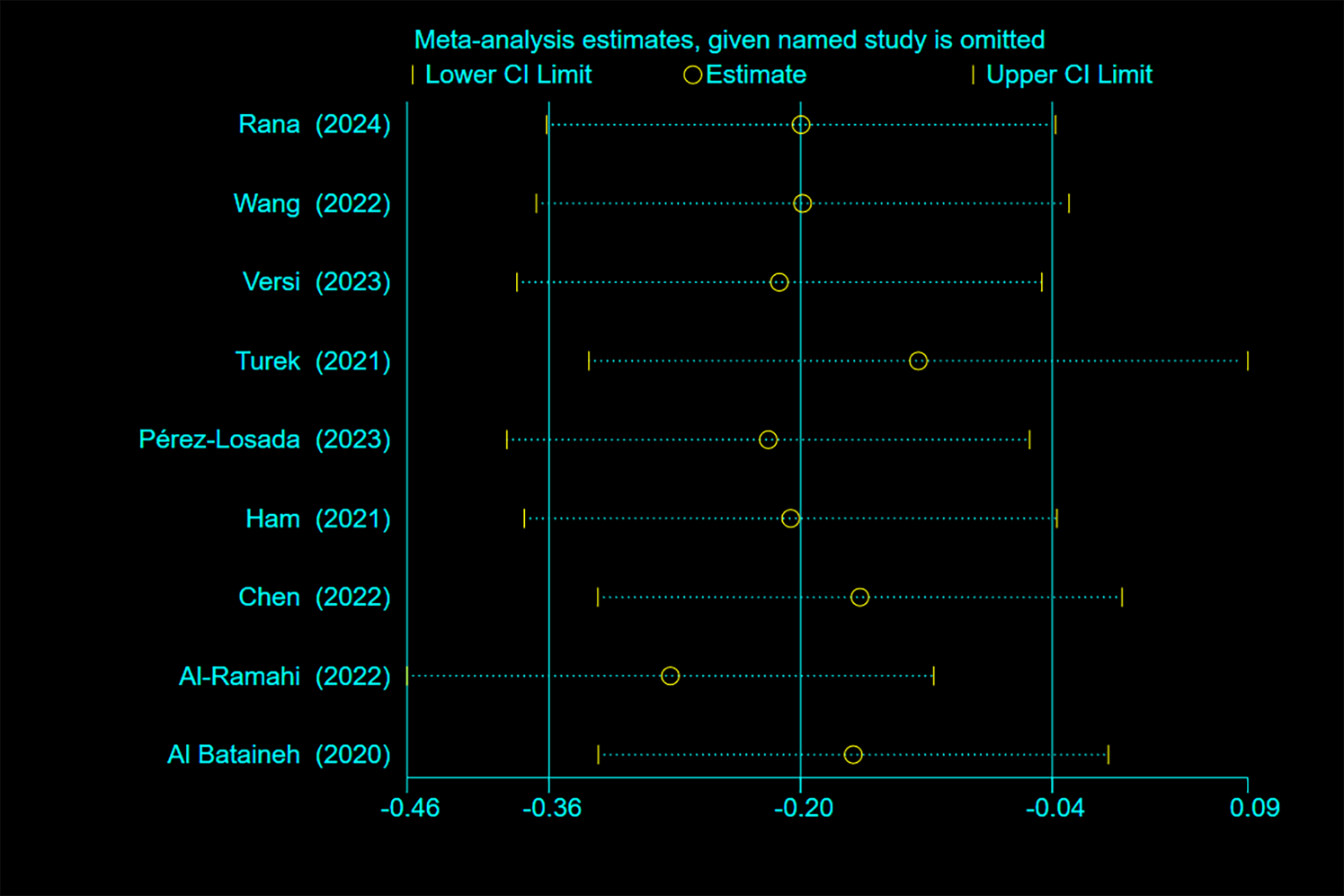
**

**Supplementary Figure 2. Sensitivity analysis for the Shannon index results for the asthma airway microbiomes of included studies.**

**
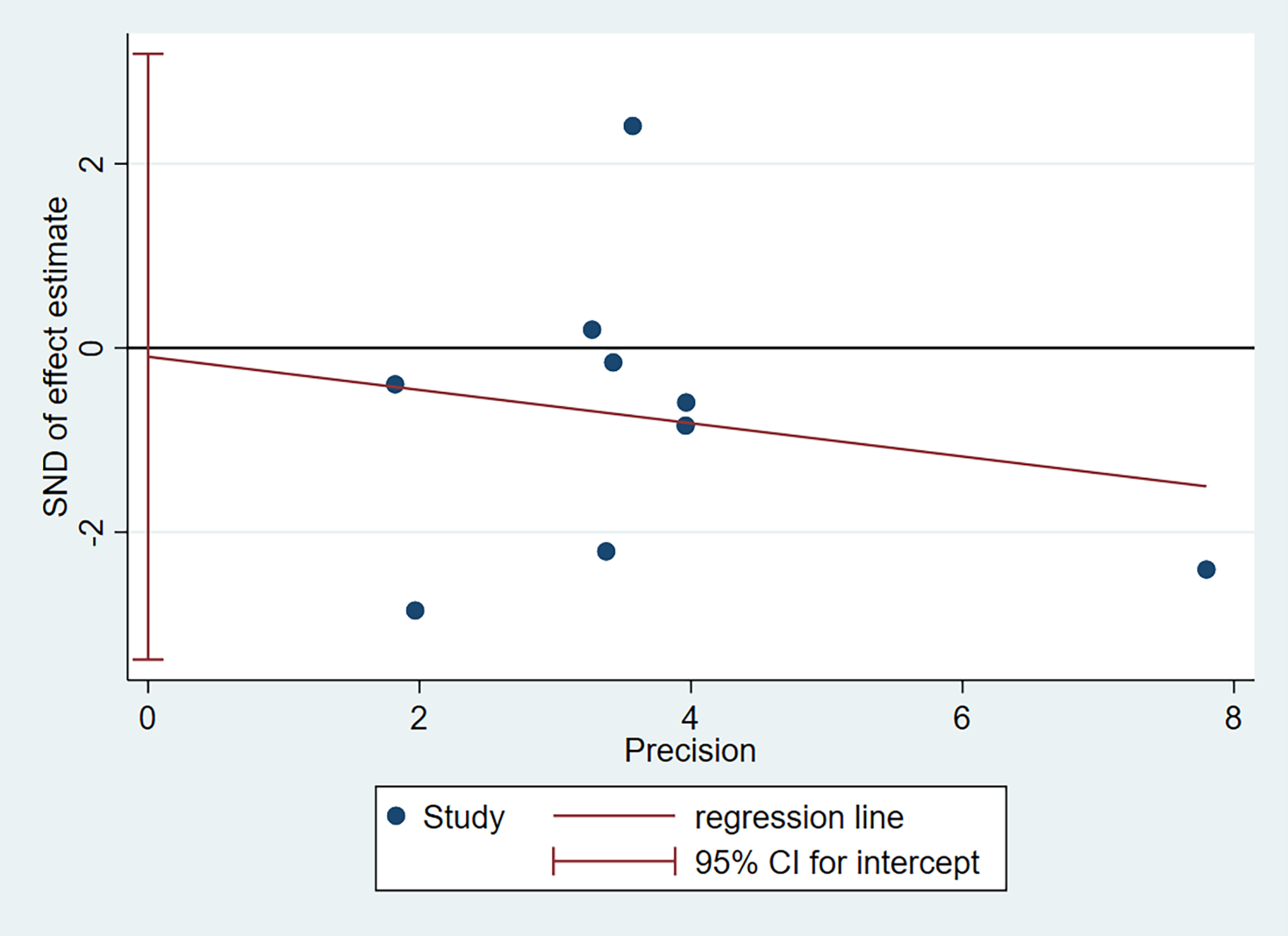
**

**Supplementary Figure 3. Egger's test for the Shannon index results for the asthma airway microbiomes of included studies.**

**
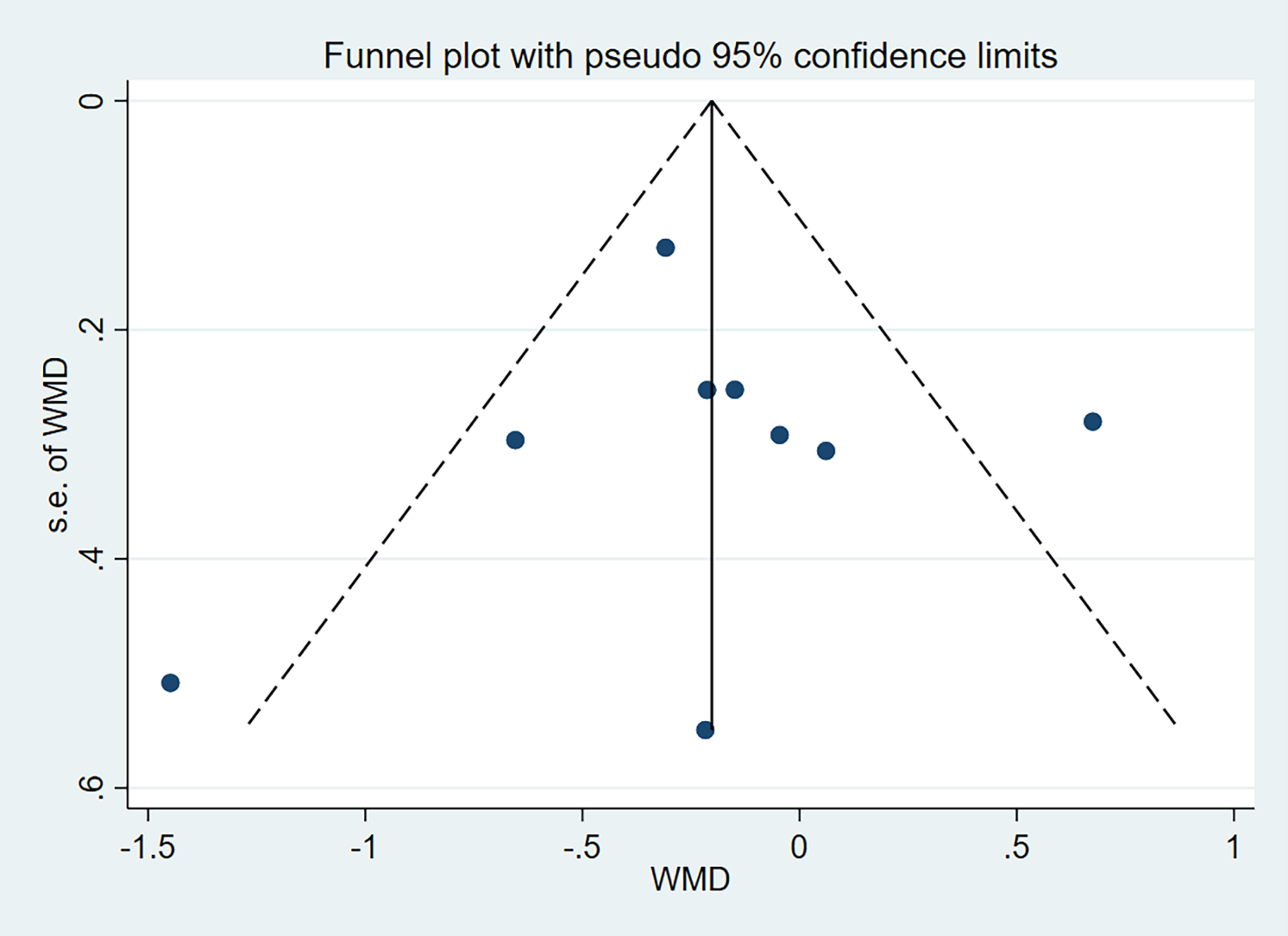
**

**Supplementary Figure 4. Funnel plot for the Shannon index results for the asthma airway microbiomes of included studies.**
